# Socioeconomic Inequalities in Oral Health among Middle-Aged and Elderly Japanese: NIPPON DATA2010

**DOI:** 10.2188/jea.JE20170247

**Published:** 2018-03-05

**Authors:** Keiko Murakami, Takayoshi Ohkubo, Mieko Nakamura, Toshiharu Ninomiya, Toshiyuki Ojima, Kayoko Shirai, Tomomi Nagahata, Aya Kadota, Nagako Okuda, Nobuo Nishi, Tomonori Okamura, Hirotsugu Ueshima, Akira Okayama, Katsuyuki Miura

**Affiliations:** 1 Department of Hygiene and Public Health, Teikyo University School of Medicine, Tokyo, Japan; 2 Department of Community Health and Preventive Medicine, Hamamatsu University School of Medicine, Shizuoka, Japan; 3 Department of Epidemiology and Public Health, Graduate School of Medical Sciences, Kyushu University, Fukuoka, Japan; 4 Department of Clinical Nursing, Shiga University of Medical Science, Shiga, Japan; 5 Department of Nutrition, School of Health and Nutrition, Tokaigakuen University, Aichi, Japan; 6 Department of Public Health, Shiga University of Medical Science, Shiga, Japan; 7 Center for Epidemiologic Research in Asia, Shiga University of Medical Science, Shiga, Japan; 8 Department of Health and Nutrition, University of Human Arts and Sciences, Saitama, Japan; 9 International Center for Nutrition and Information, National Institute of Health and Nutrition, National Institutes of Biomedical Innovation, Health and Nutrition, Tokyo, Japan; 10 Department of Preventive Medicine and Public Health, Keio University School of Medicine, Tokyo, Japan; 11 Research Institute of Strategy for Prevention, Tokyo, Japan

**Keywords:** socioeconomic status, oral health, Japan

## Abstract

**Background:**

Most studies on socioeconomic inequalities in oral health have not considered the effects of behavioral and biological factors and age differences. Furthermore, the nationwide status of inequalities remains unclear in Japan.

**Methods:**

We analyzed data from 2,089 residents aged ≥40 years throughout Japan. The lowest quartile of the number of remaining teeth for each 10-year age category was defined as poor oral health. Behavioral and biological factors included smoking status, obesity, diabetes mellitus, high-sensitivity C-reactive protein, and the use of dental devices. Multiple logistic regression analyses were conducted to examine the associations of educational attainment and equivalent household expenditure (EHE) with oral health, and stratified analyses by age category were also conducted (40–64 years and ≥65 years).

**Results:**

Lower education and lower EHE were significantly associated with an increased risk of poor oral health after adjusting for age, sex, employment status, marital and living statuses, and EHE/education; the odds ratio for junior high school education compared with ≥college education was 1.84 (95% confidence interval [CI], 1.36–2.49), and the odds ratio of the lowest compared with the highest EHE quartile was 1.91 (95% CI, 1.43–2.56). Further adjustments for behavioral and biological factors attenuated but did not eliminate these associations. EHE was significantly associated with oral health among elderly adults only, with a significant interaction by age category.

**Conclusions:**

Those with a lower education and those with lower EHE had a significantly higher risk of poor oral health, even after adjustments for behavioral and biological factors.

## INTRODUCTION

Oral health is integral and essential to quality of life and social functioning.^1^ Moreover, oral health has a profound effect on general health,^1^ as evidenced by the association between poor oral health and chronic diseases, such as cardiovascular diseases.^2^

A large number of epidemiological studies have reported an association between socioeconomic status (SES) and oral health in developed countries; lower socioeconomic groups are more likely than higher ones to have poor oral health.^3^^,^^4^ Public health research into socioeconomic inequalities in oral health has suggested causal pathways linking behavioral and biological factors to oral health.^5^ However, very few studies have examined the effects of these factors, particularly physiological markers, on the association between SES and oral health.^6^^–^^10^ Therefore, identifying the underlying causes may be useful for effective action to tackle oral health inequalities.

Previous research found that socioeconomic inequalities in general health manifested in different ways in different age categories.^11^ In oral health, a few studies suggested different inequalities according to age categories^12^^–^^15^; however, it is possible that these differences may depend on the measurements used.

There is increasing evidence to show that socioeconomic inequalities in oral health also exist in Japan,^16^^–^^20^ in which most dental as well as medical care is universally covered by the public health insurance system and tax transfers.^21^ As of 2010, the co-payment rate in Japan was set at 30%, and reduced to 10% for people aged ≥70 years.^21^ Since these studies were mainly conducted in limited areas and age groups, the nationwide status of inequalities in Japan remains unclear. The nationwide study linking the Survey of Dental Diseases and Comprehensive Survey of Living Conditions in 2005 recently showed that lower equivalent household expenditure (EHE; calculated as household expenditure divided by the square root of the number of family members) was associated with an increased risk of poor oral health.^22^ However, that study did not examine the association with education. Although different SES indicators, such as education and income, reflect a central dimension of social stratification, each represents different meanings in society.^11^^,^^23^

Therefore, the objective of the present study was to examine associations of education and economic status with oral health, with a focus on the effects of behavioral and biological factors and age differences, in a nationwide Japanese general population.

## METHODS

### Study population

In 2010, a prospective cohort study on cardiovascular diseases, the National Integrated Project for Prospective Observation of Non-communicable Disease and its Trends in the Aged 2010 (NIPPON DATA2010), was established.^24^ The study was performed with the National Health and Nutrition Survey in November 2010 (NHNS2010) and the Comprehensive Survey of Living Conditions in June 2010 (CSLC2010), which were conducted by the Ministry of Health, Labour and Welfare of Japan. The details of NHNS2010 and CSLC2010 have been described elsewhere.^25^^–^^29^

In November 2010, 8,815 residents aged ≥1 year from 300 randomly selected districts throughout Japan participated in the dietary survey for NHNS2010. Among 7,229 participants aged ≥20 years, 3,873 (1,598 men and 2,275 women) had a blood test, and 2,898 (1,239 men and 1,659 women) agreed to participate in the baseline survey of NIPPON DATA2010, which also included an electrocardiographic analysis, urinalysis, and a questionnaire on cardiovascular diseases. Trained interviewers obtained informed consent before the enrollment of study participants. The Institutional Review Board of Shiga University of Medical Science (No. 22–29, 2010) approved this study.

Of 2,898 participants, 91 were excluded because it was not possible to merge the data from NHNS2010 or CSLC2010 with NIPPON DATA2010 baseline data, and 451 aged <40 years were excluded. Of the remaining 2,356 participants aged ≥40 years, 267 were excluded because of missing data on the number of remaining teeth, educational attainment, EHE, type of house, employment status, marital and living statuses, smoking status, obesity, diabetes mellitus, high-sensitivity C-reactive protein (hs-CRP), or the use of dental devices. The remaining 2,089 participants aged ≥40 years (919 men and 1,170 women) were included in the present study. The characteristics of 267 participants who were excluded from the analysis and 2,089 analytic participants were shown in eTable 1.

### Oral health

**Figure 1.**
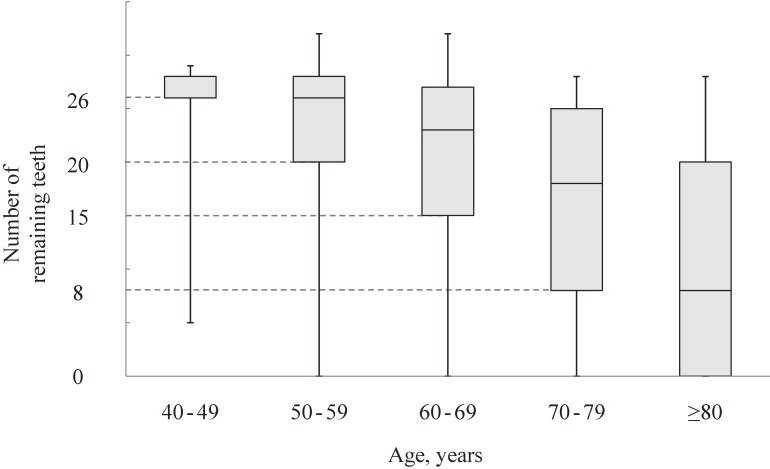
The distribution of the number of remaining teeth for each 10-year age category: NIPPON DATA2010. The lower and upper sides of the box indicate the first and third quartiles, respectively. The line that divides the box into two parts shows the median. The lower and upper whiskers indicate the locations of the minimum and maximum, respectively.

The number of remaining teeth, which was assessed for NHNS2010, was used as an indicator of oral health. Tooth loss mainly reflects a history of periodontal disease and the accumulation of caries.^30^^,^^31^ The number of remaining teeth was ascertained according to responses to the question, “How many teeth do you have? Wisdom teeth, dentures, dental bridges, and dental implants are not included. Post crowns are included.” Since the number of remaining teeth decreases with advancing age, we decided to set different cut-off points of poor oral health for each age group. The lowest quartile of the number of remaining teeth for each 10-year age group was employed as the definition of poor oral health; ≤26 teeth, ≤20 teeth, ≤15 teeth, ≤8 teeth, and 0 teeth among those aged 40–49, 50–59, 60–69, 70–79, and ≥80 years, respectively (Figure 1).

### SES

Based on the self-administered questionnaire for NIPPON DATA2010, participants were sorted into three categories of educational attainment: junior high school, high school, college or higher (college [including special training school], university, or graduate school). Monthly EHE of May 2010, the month before CSLC2010, was collected from the self-administered questionnaire for CSLC2010, and participants were grouped by quartiles of expenditure. Annual household income was also obtained from the self-administered questionnaire for NHNS2010 (<2, 2–6, or ≥6 million Japanese yen [JPY]).

### Covariates

The type of house (owned or rented) was obtained using the questionnaire for CSLC2010 and was used as a covariate as EHE that included household rent, but not a mortgage. Employment status (employed [including self-employed] or unemployed) was obtained using the questionnaire for CSLC2010. Marital and living statuses were divided into married, unmarried (including never married, divorced, and widowed) and not living alone, or unmarried and living alone, using the questionnaire for NIPPON DATA2010.

Behavioral and biological factors were smoking status, obesity, diabetes mellitus, hs-CRP, and the use of dental devices. Public health nurses collected information on the smoking status, which was classified into never, former, or current. Body mass index (BMI) was calculated as body weight (kg) divided by the square of height (m^2^), measured with participants in light clothing without shoes for NHNS2010. Obesity was defined as BMI ≥25 kg/m^2^, adopting the definition of the Japan Society for the Study of Obesity.^32^ Casual blood samples were obtained for NHNS2010. If a blood sample was taken after ≥8 hours of fasting, it was defined as a fasting blood sample. Participants were classified as diabetic if they met one of the following criteria^33^: fasting plasma glucose level of ≥126 mg/dL (≥7.0 mmol/L); non-fasting plasma glucose level of ≥200 mg/dL (≥11.1 mmol/L); HbA1c ≥6.5%; and/or the use of antidiabetic medications. A hs-CRP level of >0.1 mg/dL was considered to be elevated, according to a Japanese study.^34^ The use of dental devices was ascertained from the questionnaire for NHNS2010 by asking, “Do you use any of the following devices (devices for promoting oral health): tooth brush; interdental brush; tongue brush; dental floss/floss pick.” If participants used at least one of interdental brush, tongue brush, or dental floss/floss pick, they were considered to be users.

### Statistical analysis

The characteristics of participants aged 40–64 years and ≥65 years were compared using the Student’s *t*-test for continuous variables and the chi-squared test for categorical variables. Multiple logistic regression analyses were conducted to examine associations of education and EHE with oral health. In model 1, we calculated the odds ratio (OR) and 95% confidence interval (CI) of education or EHE adjusted for age and sex (and the type of house in the analysis of EHE only). We made further adjustments for employment status, marital and living statuses, and EHE/education (model 2), as well as behavioral and biological factors (model 3). We also stratified our models by age category (40–64 years and ≥65 years) and examined whether it modified the associations of education and EHE with oral health by including interaction terms in the models.

Sensitivity analyses were conducted using annual household income classification instead of EHE and further adjusting for the square root of the number of family members. We also conducted sex-stratified analyses and examined the interaction between sex and education/EHE.

All analyses were conducted using Stata 12.0 (StataCorp LP, College Station, TX, USA). In all analyses, a two-tailed *P* < 0.05 was considered to be significant.

## RESULTS

Table 1 shows the characteristics of the study participants. The mean age of 2,089 participants was 63.6 (standard deviation [SD], 11.4) years, 56.0% were women, and 26.8% were educated beyond high school. Significant differences in characteristics were observed between those aged 40–64 years and those aged ≥65 years, except for sex, EHE, and obesity. The median number of remaining teeth was 23 (interquartile range, 14–27); 26 teeth (interquartile range, 20–28) among those aged 40–64 years and 19 teeth (interquartile range, 8–25) among those aged ≥65 years.

**Table 1.** Characteristics of study participants: NIPPON DATA2010

	Total (*N* = 2,089)	Age category		*P* value^a^

		40–64 years (*n* = 1,052)	≥65 years (*n* = 1,037)	
Age, years, mean (SD)	63.6 (11.4)	54.4 (7.2)	73.0 (5.9)	<0.001
Women, *n* (%)	1,170 (56.0)	603 (57.3)	567 (54.7)	0.224
Educational attainment, *n* (%)				<0.001
College or higher	560 (26.8)	395 (37.5)	165 (15.9)	
High school	953 (45.6)	509 (48.4)	444 (42.8)	
Junior high school	576 (27.6)	148 (14.1)	428 (41.3)	
EHE,^b^ mean (SD)	148.8 (114.6)	151.6 (127.0)	146.0 (100.5)	0.265
Owned a house, *n* (%)	1,790 (85.7)	882 (83.8)	908 (87.6)	0.015
Employed, *n* (%)	1,010 (48.4)	748 (71.1)	262 (25.3)	<0.001
Marital and living statuses, *n* (%)				<0.001
Married	1,662 (79.5)	878 (83.5)	784 (75.6)	
Single, not living alone	202 (9.7)	103 (9.8)	99 (9.6)	
Single, living alone	225 (10.8)	71 (6.7)	154 (14.8)	
Smoking status, *n* (%)				<0.001
Never smoker	1,377 (65.9)	664 (63.1)	713 (68.8)	
Former smoker	412 (19.7)	174 (16.5)	238 (22.9)	
Current smoker	300 (14.4)	214 (20.4)	86 (8.3)	
Obesity (BMI ≥25.0 kg/m^2^), *n* (%)	588 (28.2)	294 (28.0)	294 (28.4)	0.837
Diabetes mellitus, *n* (%)	240 (11.5)	89 (8.5)	151 (14.6)	<0.001
Elevated hs-CRP (>0.1 mg/dL), *n* (%)	449 (21.5)	194 (18.4)	255 (24.6)	0.001
Use of dental devices, *n* (%)	769 (36.8)	416 (39.5)	353 (34.0)	0.009
Number of remaining teeth, median (interquartile range)	23 (14–27)	26 (20–28)	19 (8–25)	
0 teeth, *n* (%)	159 (7.6)	22 (2.1)	137 (13.2)	<0.001
1–9 teeth, *n* (%)	201 (9.6)	50 (4.7)	151 (14.6)	
10–19 teeth, *n* (%)	382 (18.3)	148 (14.1)	234 (22.6)	
20–24 teeth, *n* (%)	425 (20.4)	210 (20.0)	215 (20.7)	
≥25 teeth, *n* (%)	922 (44.1)	622 (59.1)	300 (28.9)	

BMI, body mass index; EHE, equivalent household expenditure; hs-CRP, high-sensitivity C-reactive protein.

^a^Obtained using the Student’s *t*-test for continuous variables and the chi-squared test for categorical variables, comparing age categories.

^b^Thousand Japanese yen (/month).

Table 2 shows the prevalence, ORs, and 95% CIs of poor oral health. Lower educational attainment and lower EHE were significantly associated with an increased risk of poor oral health after adjusting for age, sex, type of house, employment status, marital and living statuses, and EHE/education (model 2); the multivariable-adjusted OR of junior high school education compared with college education or higher was 1.84 (95% CI, 1.36–2.49), and the multivariable-adjusted OR of the lowest compared with the highest EHE quartile was 1.91 (95% CI, 1.43–2.56). After further adjustments for behavioral and biological factors, this association was somewhat attenuated but did not disappear (model 3); the corresponding ORs were 1.37 (95% CI, 1.01–1.88) and 1.75 (95% CI, 1.30–2.37), respectively. Current smoking, obesity, diabetes mellitus, elevated hs-CRP, and the non-use of dental devices were significantly associated with an increased risk of poor oral health after adjusting for age and sex. After multivariate adjustments, the associations of current smoking and diabetes mellitus with oral health remained significant.

**Table 2.** Associations of educational attainment and EHE with poor oral health: NIPPON DATA2010

	Poor oral health/ participants	(%)	Model 1	Model 2	Model 3
			OR (95% CI)	OR (95% CI)	OR (95% CI)
Educational attainment					
College or higher	114/560	(20.4)	1.00	1.00	1.00
High school	255/953	(26.8)	1.50 (1.16–1.94)	1.43 (1.10–1.86)	1.25 (0.95–1.63)
Junior high school	197/576	(34.2)	2.18 (1.62–2.92)	1.84 (1.36–2.49)	1.37 (1.01–1.88)
EHE quartiles					
4th (highest)	98/472	(20.8)	1.00	1.00	1.00
3rd	137/571	(24.0)	1.18 (0.88–1.59)	1.15 (0.85–1.55)	1.09 (0.80–1.48)
2nd	139/521	(26.7)	1.36 (1.01–1.83)	1.26 (0.93–1.70)	1.20 (0.88–1.63)
1st (lowest)	192/525	(36.6)	2.16 (1.62–2.88)	1.91 (1.43–2.56)	1.75 (1.30–2.37)
Covariates					
Age (per 10-year increase)			1.06 (0.97–1.15)	0.95 (0.86–1.06)	0.98 (0.87–1.09)
Sex					
Men	277/919	(30.1)	1.00	1.00	1.00
Women	289/1170	(24.7)	0.76 (0.63–0.93)	0.72 (0.58–0.88)	1.13 (0.86–1.49)
Type of house					
Owned a house	470/1790	(26.3)	1.00	1.00	1.00
Rented a house	96/299	(32.1)	1.36 (1.04–1.77)	1.21 (0.92–1.61)	1.26 (0.94–1.68)
Employment status					
Employed	269/1010	(26.6)	1.00	1.00	1.00
Unemployed	297/1079	(27.5)	1.05 (0.83–1.32)	1.06 (0.84–1.34)	1.09 (0.86–1.38)
Marital and living statuses					
Married	428/1662	(25.8)	1.00	1.00	1.00
Single, not living alone	64/202	(31.7)	1.41 (1.03–1.95)	1.32 (0.95–1.83)	1.16 (0.83–1.62)
Single, living alone	74/225	(32.9)	1.44 (1.06–1.96)	1.27 (0.92–1.75)	1.11 (0.80–1.56)
Smoking status					
Never smoker	327/1377	(23.8)	1.00		1.00
Former smoker	121/412	(29.4)	1.34 (1.00–1.80)		1.31 (0.96–1.78)
Current smoker	118/300	(39.3)	2.24 (1.65–3.05)		1.94 (1.40–2.69)
Obesity (BMI ≥25.0 kg/m^2^)					
No	380/1501	(25.3)	1.00		1.00
Yes	186/588	(31.6)	1.32 (1.07–1.63)		1.15 (0.92–1.44)
Diabetes mellitus					
No	476/1849	(25.7)	1.00		1.00
Yes	90/240	(37.5)	1.66 (1.25–2.21)		1.47 (1.09–1.98)
Elevated hs-CRP (>0.1 mg/dL)					
No	419/1640	(25.6)	1.00		1.00
Yes	147/449	(32.7)	1.37 (1.09–1.72)		1.15 (0.90–1.46)
Use of dental devices					
No	456/1320	(34.6)	1.00		1.00
Yes	110/769	(14.3)	0.32 (0.26–0.41)		0.36 (0.28–0.46)

BMI, body mass index; CI, confidence interval; EHE, equivalent household expenditure; hs-CRP, high-sensitivity C-reactive protein; OR, odds ratio.

Model 1: adjusted for age (per 10-year increase), sex, and type of house (own or rent: in the analysis of EHE only).

Model 2: Model 1 + adjusted for employment status, marital and living statuses, and EHE quartiles/educational attainment.

Model 3: Model 2 + adjusted for smoking status, obesity, diabetes mellitus, elevated hs-CRP, and the use of dental devices.

Table 3 shows the results of stratified analyses by age categories. Educational attainment was significantly associated with oral health only among those aged 40–64 years, after adjusting for age, sex, employment status, marital and living statuses, EHE, and type of house (model 2); however, no significant interaction was found between age category and educational attainment (*P* = 0.51). EHE was significantly associated with oral health only among those aged ≥65 years after adjusting for age, sex, employment status, marital and living statuses, education, and type of house (model 2), with a significant interaction by the age category (*P* = 0.020). These associations did not materially change after further adjustments for behavioral and biological factors (model 3). The associations of behavioral and biological factors with oral health by age categories are shown in eTable 2.

**Table 3.** Associations of educational attainment and EHE with poor oral health according to age categories: NIPPON DATA2010

	Poor oral health/ participants	(%)	Model 1	Model 2	Model 3
			OR (95% CI)	OR (95% CI)	OR (95% CI)
Educational attainment					
40–64 years (*n* = 1,052)					
College or higher	77/395	(19.5)	1.00	1.00	1.00
High school	148/509	(29.1)	1.85 (1.34–2.55)	1.84 (1.33–2.56)	1.59 (1.14–2.23)
Junior high school	53/148	(35.8)	2.71 (1.73–4.24)	2.42 (1.52–3.84)	1.74 (1.07–2.82)
≥65 years (*n* = 1,037)					
College or higher	37/165	(22.4)	1.00	1.00	1.00
High school	107/444	(24.1)	1.12 (0.73–1.73)	1.00 (0.64–1.56)	0.90 (0.56–1.43)
Junior high school	144/428	(33.6)	1.70 (1.12–2.60)	1.35 (0.87–2.09)	1.01 (0.63–1.60)

EHE quartiles					
40–64 years (*n* = 1,052)					
4th (highest)	60/243	(24.7)	1.00	1.00	1.00
3rd	67/289	(23.2)	0.93 (0.62–1.38)	0.87 (0.58–1.31)	0.85 (0.56–1.29)
2nd	62/265	(23.4)	0.91 (0.60–1.37)	0.82 (0.54–1.25)	0.82 (0.53–1.25)
1st (lowest)	89/255	(34.9)	1.57 (1.06–2.32)	1.33 (0.88–1.99)	1.26 (0.83–1.92)
≥65 years (*n* = 1,037)					
4th (highest)	38/229	(16.6)	1.00	1.00	1.00
3rd	70/282	(24.8)	1.61 (1.04–2.51)	1.61 (1.03–2.52)	1.52 (0.95–2.41)
2nd	77/256	(30.1)	2.14 (1.37–3.32)	2.02 (1.29–3.17)	1.92 (1.21–3.05)
1st (lowest)	103/270	(38.2)	3.03 (1.97–4.66)	2.81 (1.81–4.36)	2.71 (1.72–4.27)

CI, confidence interval; EHE, equivalent household expenditure; OR, odds ratio.

Interaction between the age category and educational attainment: Model 1, *P* = 0.557; Model 2, *P* = 0.513; Model 3, *P* = 0.715.

Interaction between the age category and EHE quartiles: Model 1, *P* = 0.019; Model 2, *P* = 0.020; Model 3, *P* = 0.012.

Model 1: adjusted for age (per 10-year increase), sex, and type of house (own or rent: in the analysis of EHE only).

Model 2: Model 1 + adjusted for employment status, marital and living statuses, and EHE quartiles/educational attainment.

Model 3: Model 2 + adjusted for smoking status, obesity (body mass index ≥25.0 kg/m^2^), diabetes mellitus, elevated high-sensitivity C-reactive protein (>0.1 mg/dL), and the use of dental devices.

Sensitivity analyses using the annual household income classification instead of EHE also revealed a significant association between household income and oral health; the multivariable-adjusted OR of those with a household income of <2 million JPY compared with those with ≥6 million JPY was 1.92 (95% CI, 1.32–2.80) (model 3) (eTable 3).

Sensitivity analyses stratified by sex showed that educational attainment was significantly associated with oral health among women only after adjusting for age, employment status, marital and living statuses, EHE, and the type of house (model 2); however, no significant interaction was found between sex and educational attainment (*P* = 0.29) (eTable 4). EHE was significantly associated with oral health among men and women.

## DISCUSSION

In the present analysis of a nationwide survey of the general Japanese population, those with a lower education and those with lower EHE had a significantly higher risk of poor oral health. These associations were attenuated but did not disappear after further adjustments for behavioral and biological factors. Lower EHE was significantly associated with an increased risk of poor oral health among elderly, not middle-aged adults, with a significant interaction by age category.

A lower education was associated with an increased risk of poor oral health independent of EHE. Education may convey specific factual knowledge about health and raise cognitive skills that foster health-promoting decisions.^11^^,^^35^^,^^36^ Education also shapes cultural capital,^37^ which takes the form of health-related values and norms.^38^^,^^39^ Since cultural values related to oral health are known to influence the adoption of efficacious preventive behaviors,^40^ the unequal distribution of cultural capital across educational levels may result in oral health inequalities. Social networks, which combine individual resources with those of others,^41^ may also partially explain educational inequalities in oral health. Education increases the chance to associate with other highly-educated individuals, and their social networks promote health and widen inequalities.^36^^,^^41^^,^^42^ Since several studies have reported an association between social networks and oral health,^43^ the potential role of social networks in health inequalities may also be applied to oral health.

Lower EHE was significantly associated with an increased risk of poor oral health independent of educational attainment. Some Japanese studies also found income-related inequalities in oral health^19^^,^^20^ and EHE-related inequalities in oral health.^22^ Socioeconomic inequalities in oral health are sometimes attributed to large sections of adult dental care being excluded from public care insurance packages in many countries.^7^^,^^10^ It is noteworthy that economic status is a major factor affecting oral health in Japan, in which universal health insurance covers most dental care.

A higher economic status may promote the adoption of commodities for enhancing oral health. Our results showed that use of dental devices, which is one form of dental self-care, led to a significantly lower risk of poor oral health, whereas the significant association between EHE and oral health remained even after adjusting for the use of dental devices. One study in Australia showed that the magnitude of socioeconomic inequalities in oral health was significantly attenuated by dental visits but not by dental self-care.^6^ We previously identified income-related inequality in dental visits for preventive purposes (dental scaling, fluoride, or orthodontic treatments), not for curative purposes among working-age Japanese men.^44^ Dental care in Japan has traditionally been treatment-oriented, and the prevalence of preventive dental care remains relatively low.^28^^,^^44^ There are extensive prevention possibilities, and prevention may actually save resources, particularly in the case of oral health,^45^ suggesting that inequalities in preventive dental visits but not dental self-care partly explain economic inequalities in oral health, as demonstrated in the present study. Since the prevention of tooth loss may help maintain a high quality of life and good general health,^1^ measures need to take advantage of preventive techniques.

Current smoking, obesity, diabetes mellitus, elevated hs-CRP, and the non-use of dental devices were significantly associated with an increased risk of poor oral health after adjusting for age and sex. The associations between SES and oral health were somewhat attenuated but did not disappear after further adjustments for these behavioral and biological factors. This attenuation suggests that improvements in these factors lessen the magnitude of socioeconomic inequalities in oral health, although we cannot reach any concrete conclusion on the causal direction due to the cross-sectional design. Furthermore, the remaining associations indicate that these factors are insufficient to explain these inequalities, which is consistent with the findings of previous studies using health behaviors^6^^–^^8^^,^^10^ and physiological markers.^9^ These results suggest the presence of more complex determinants of oral health inequalities and imply that the elimination of oral health inequalities necessitates the development of strategies that look beyond proximal causes, such as early-life unequal exposure to risk factors.^31^

In the present study, current smokers and participants with diabetes mellitus had a significantly higher risk of poor oral health, even after multivariate adjustments. Evidence supporting the causal association between smoking and tooth loss is consistently strong,^46^ and a large number of studies have demonstrated that diabetes mellitus is associated with an increased risk of periodontitis,^47^ which is an important cause of tooth loss as well as dental caries.^30^^,^^31^ The present study confirmed these findings.

Lower EHE was significantly associated with oral health only among those aged ≥65 years, with a significant interaction by the age category. Previous studies found that the magnitude of income-related inequalities in the number of remaining teeth became progressively stronger with advancing age.^12^^,^^13^^,^^15^ These findings are consistent with the present results. In contrast, other studies showed that the association between income and subjective oral health became progressively weaker with advancing age.^12^^–^^14^ Tooth loss is a consequence of cumulative factors over the course of an individual’s lifetime.^31^ Although variations in oral health inequalities according to age categories are still unclear partly due to the small number of studies conducted, previous studies^12^^,^^13^^,^^15^ and our present results consistently suggested that the magnitude of socioeconomic inequalities in the number of remaining teeth increased with advancing age. The number of remaining teeth may be a good reflection of inequality that has emerged toward the end of life, but is not useful early in life.

Some limitations of the present study need to be discussed. Since this was a cross-sectional study, we were unable to confirm the causal direction of the associations observed. However, poor oral health in adulthood is unlikely to affect educational levels. Furthermore, since SES indicators were self-reported, these may be subject to bias due to under- or over-reporting. In addition, the measures of oral health were also self-reported, without clinical examinations. However, previous studies in Japan confirmed the validity of the self-reported number of remaining teeth among middle-aged^48^ and elderly adults^49^ in a general population. The number of remaining teeth may have been underestimated among adults who underwent dental implant therapy.

In conclusion, the present analysis of a nationwide survey of the general Japanese population demonstrates that those with a lower education and those with lower EHE had a significantly higher risk of poor oral health. These associations were attenuated but did not disappear after further adjustments for behavioral and biological factors. Lower EHE was significantly associated with an increased risk of poor oral health among elderly, not middle-aged adults, with a significant interaction by age category. Further research is needed in order to elucidate the mechanisms by which SES affects oral health. These results will contribute to orienting public health intervention initiatives that reduce socioeconomic inequalities in oral health.

## APPENDIX A. SUPPLEMENTARY DATA

eTable 1. Characteristic differences between 2,089 participants who were analyzed and 267 participants who were excluded from the analysis: NIPPON DATA2010

eTable 2. Associations of behavioral and biological factors with poor oral health according to age categories: NIPPON DATA2010

eTable 3. Association between household income and poor oral health: NIPPON DATA2010

eTable 4. Associations of educational attainment and EHE with poor oral health by sex: NIPPON DATA2010
