# Supplementary material for: Socioeconomic Inequalities in Oral Health among Middle-Aged and Elderly Japanese: NIPPON DATA2010
Source: J Epidemiol. 2018 Mar 5;28(Suppl 3):S59–65. doi: 10.2188/jea.JE20170247 (PMC5825696; doi:10.2188/jea.JE20170247)
Supplement: Supplementary file 1 [file je-28-S059-s001.pdf]

eTable 1. Characteristic differences between 2,089 participants who were analyzed and 267 participants who were excluded from the analysis: NIPPON DATA2010

	Participants who were analyzed (n=2,089)		Participants who were not analyzed (n=267)		*P* value[a]
Age, years, mean (SD)	63.6	(11.4)	67.3	(11.2)	<0.001
Women, n (%)	1,170	(56.0)	138	(51.7)	0.181
Educational attainment, n (%)					0.015
College or higher	560	(26.8)	57	(21.7)	
High school	953	(45.6)	111	(42.4)	
Junior high school	576	(27.6)	94	(35.9)	
EHE,[b] mean (SD)	148.8	(114.6)	137.8	(101.4)	0.258
Owned a house, n (%)	1,790	(85.7)	196	(73.4)	<0.001
Employed, n (%)	1,010	(48.4)	83	(44.4)	0.299
Marital and living statuses, n (%)					0.036
Married	1,662	(79.5)	182	(72.5)	
Single, not living alone	202	(9.7)	33	(13.2)	
Single, living alone	225	(10.8)	36	(14.3)	
Smoking status, n (%)					0.409
Never smoker	1,377	(65.9)	163	(63.2)	
Former smoker	412	(19.7)	60	(23.2)	
Current smoker	300	(14.4)	35	(13.6)	
Obesity (BMI ≥25.0 kg/m^2), n (%)	588	(28.2)	101	(37.8)	0.001
Diabetes mellitus, n (%)	240	(11.5)	79	(29.6)	<0.001
Elevated hs-CRP (>0.1 mg/dL), n (%)	449	(21.5)	49	(22.9)	0.635
Use of dental devices, n (%)	769	(36.8)	84	(32.2)	0.143
Number of remaining teeth, median (interquartile range)	23	(14–27)	20	(9–25)	
0 teeth, n (%)	159	(7.6)	28	(10.9)	<0.001
1–9 teeth, n (%)	201	(9.6)	40	(15.5)	
10–19 teeth, n (%)	382	(18.3)	58	(22.6)	
20–24 teeth, n (%)	425	(20.4)	58	(22.6)	
≥25 teeth, n (%)	922	(44.1)	73	(28.4)	

BMI, body mass index; EHE, equivalent household expenditure; hs-CRP, high-sensitivity C-reactive protein.

Percentages are shown after excluding those whose values were missing.

[a] Obtained using the Student's *t*-test for continuous variables and the chi-squared test for categorical variables, comparing participants who were analyzed and those who were not.

[b] Thousand Japanese yen (/month).

eTable 2. Associations of behavioral and biological factors with poor oral health according to age categories: NIPPON DATA2010

	40–64 years (n=1,052)			≥65 years (n=1,037)			*P* for interactions[a]
	Poor oral health / participants	(%)	OR (95% CI)	Poor oral health / participants	(%)	OR (95% CI)	
Smoking status							0.738
Never smoker	140 /664	(21.1)	1.00	187 / 713	(26.2)	1.00	
Former smoker	55 / 174	(31.6)	1.52 (0.96–2.38)	66 / 238	(27.7)	1.29 (0.84–1.99)	
Current smoker	83 / 214	(38.8)	1.82 (1.20–2.75)	35 / 86	(40.7)	2.36 (1.35–4.10)	
Obesity (BMI ≥25.0 kg/m^2)							0.110
No	177 / 758	(23.4)	1.00	203 / 743	(27.3)	1.00	
Yes	101 / 294	(34.4)	1.32 (0.95–1.82)	85 / 294	(28.9)	0.97 (0.70–1.34)	
Diabetes mellitus							0.370
No	246 / 963	(25.6)	1.00	230 / 886	(26.0)	1.00	
Yes	32 / 89	(36.0)	1.15 (0.70–1.90)	58 / 151	(38.4)	1.77 (1.19–2.62)	
Elevated hs-CRP (>0.1 mg/dL)							0.009
No	203 / 858	(23.7)	1.00	216 / 782	(27.6)	1.00	
Yes	75 / 194	(38.7)	1.62 (1.13–2.31)	72 / 255	(28.2)	0.83 (0.58–1.17)	
Use of dental devices							0.010
No	209 / 636	(32.9)	1.00	247 / 684	(36.1)	1.00	
Yes	69 / 416	(16.6)	0.50 (0.36–0.69)	41 / 353	(11.6)	0.26 (0.18–0.37)	

BMI, body mass index; CI, confidence interval; hs-CRP, high-sensitivity C-reactive protein; OR, odds ratio.

[a] *P* denotes the significance of the interaction between age categories (40–64 years, ≥65 years) and each variable.

Adjusted for age (per 10-year increase), sex, type of house, employment status, marital and living statuses, educational attainment, equivalent household expenditure quartiles, and smoking status/obesity/diabetes mellitus/elevated hs-CRP/the use of dental devices.

eTable 3. Association between household income and poor oral health: NIPPON DATA2010

	Poor oral health / participants	(%)	Model 1 OR (95% CI)	Model 2 OR (95% CI)	Model 3 OR (95% CI)
Total (N=1,894)					
≥6 million JPY[a]	86 / 404	(21.3)	1.00	1.00	1.00
2–6 million JPY[a]	278 / 1091	(25.5)	1.40 (1.05–1.86)	1.30 (0.97–1.74)	1.21 (0.90–1.64)
<2 million JPY[a]	146 / 399	(36.6)	2.69 (1.90–3.82)	2.14 (1.48–3.09)	1.92 (1.32–2.80)
40–64 years (n=951)					
≥6 million JPY[a]	59 / 296	(19.9)	1.00	1.00	1.00
2–6 million JPY[a]	149 / 518	(28.8)	1.78 (1.25–2.53)	1.56 (1.08–2.25)	1.47 (1.01–2.14)
<2 million JPY[a]	45 / 137	(32.9)	2.25 (1.38–3.66)	1.81 (1.07–3.07)	1.57 (0.92–2.70)
≥65 years (n=943)					
≥6 million JPY[a]	27 / 108	(25.0)	1.00	1.00	1.00
2–6 million JPY[a]	129 / 573	(22.5)	1.10 (0.67–1.82)	1.13 (0.68–1.88)	1.08 (0.63–1.84)
<2 million JPY[a]	101 / 262	(38.6)	2.95 (1.67–5.20)	2.63 (1.46–4.75)	2.51 (1.35–4.66)

CI, confidence interval; OR, odds ratio.

Interaction between the age category and household income: Model 1, P=0.327; Model 2, P=0.205; Model 3, P=0.117.

[a] Japanese yen (/year).

Model 1: adjusted for age (per 10-year increase), sex, and the square root of the number of family members.

Model 2: Model 1 + adjusted for employment status, marital and living statuses, and educational attainment.

Model 3: Model 2 + adjusted for smoking status, obesity (body mass index ≥25.0 kg/m^2), diabetes mellitus, elevated high-sensitivity C-reactive protein (>0.1 mg/dL), and the use of dental devices.

eTable 4. Associations of educational attainment and EHE with poor oral health by sex: NIPPON DATA2010

	Poor oral health / participants	(%)	Model 1 OR (95% CI)	Model 2 OR (95% CI)	Model 3 OR (95% CI)
Educational attainment					
Men (n=919)					
College or higher	58 / 272	(21.3)	1.00	1.00	1.00
High school	101 / 389	(26.0)	1.29 (0.89–1.87)	1.21 (0.83–1.76)	1.06 (0.72–1.56)
Junior high school	79 / 258	(30.6)	1.62 (1.07–2.46)	1.36 (0.89–2.10)	0.99 (0.64–1.55)
Women (n=1,170)					
College or higher	55 / 288	(19.1)	1.00	1.00	1.00
High school	147 / 564	(26.1)	1.54 (1.07–2.21)	1.50 (1.03–2.16)	1.32 (0.90–1.94)
Junior high school	112 / 318	(35.2)	2.42 (1.60–3.68)	2.11 (1.37–3.24)	1.63 (1.04–2.54)
EHE quartiles					
Men (n=919)					
4th (highest)	39 / 202	(19.3)	1.00	1.00	1.00
3rd	56 / 250	(22.4)	1.19 (0.75–1.88)	1.17 (0.74–1.87)	1.11 (0.69–1.79)
2nd	63 / 233	(27.0)	1.53 (0.97–2.41)	1.49 (0.94–2.36)	1.49 (0.93–2.39)
1st (lowest)	80 / 234	(34.2)	2.17 (1.40–3.38)	2.04 (1.30–3.22)	1.89 (1.19–3.01)
Women (n=1,170)					
4th (highest)	53 / 270	(19.6)	1.00	1.00	1.00
3rd	80 / 321	(24.9)	1.35 (0.91–1.99)	1.31 (0.88–1.95)	1.28 (0.85–1.92)
2nd	74 / 288	(25.7)	1.40 (0.94–2.09)	1.29 (0.86–1.93)	1.16 (0.77–1.76)
1st (lowest)	107 / 291	(36.8)	2.32 (1.58–3.41)	2.06 (1.39–3.05)	1.89 (1.26–2.83)

CI, confidence interval; EHE, equivalent household expenditure; OR, odds ratio.

The definitions of poor oral health among men were ≤25 teeth, ≤19 teeth, ≤14 teeth, ≤7 teeth, and 0 teeth among those aged 40–49, 50–59, 60–69, 70–79, and ≥80 years, respectively. The definitions of poor oral health among women were ≤26 teeth, ≤22 teeth, ≤16 teeth, ≤8 teeth, and 0 teeth among those aged 40–49, 50–59, 60–69, 70–79, and ≥80 years, respectively.

Interaction between sex and educational attainment: Model 1, P=0.192; Model 2, P=0.285; Model 3, P=0.346.

Interaction between sex and EHE quartiles: Model 1, P=0.960; Model 2, P=0.991; Model 3, P=0.875.

Model 1: adjusted for age (per 10-year increase) and type of house (own or rent: in the analysis of EHE only).

Model 2: Model 1 + adjusted for employment status, marital and living statuses, and EHE quartiles/educational attainment.
Model 3: Model 2 + adjusted for smoking status, obesity (body mass index ≥25.0 kg/m^2), diabetes mellitus, elevated high-sensitivity C-reactive protein (>0.1 mg/dL), and the use of dental devices.
